# Evaluation of Factors Affecting Prescribing Behaviors, in Iran Pharmaceutical Market by Econometric Methods

**Published:** 2015

**Authors:** Nima Tahmasebi, Abbas Kebriaeezadeh

**Affiliations:** a *Department of Pharmacoeconomics and Pharmaceutical Administration, Faculty of Pharmacy, Shahid Beheshti University of Medical Sciences. *; b *Department of Pharmacoeconomics and Pharmaceutical Administration, Faculty of Pharmacy, Tehran University of Medical Sciences.*

**Keywords:** Advertisement, Characteristics of physicians, Price, Drug sales, Econometrics

## Abstract

Prescribing behavior of physicians affected by many factors. The present study is aimed at discovering the simultaneous effects of the evaluated factors (including: price, promotion and demographic characteristics of physicians) and quantification of these effects. In order to estimate these effects, Fluvoxamine (an antidepressant drug) was selected and the model was figured out by panel data method in econometrics. We found that insurance and advertisement respectively are the most effective on increasing the frequency of prescribing, whilst negative correlation was observed between price and the frequency of prescribing a drug. Also brand type is more sensitive to negative effect of price than to generic. Furthermore, demand for a prescription drug is related with physician demographics (age and sex). According to the results of this study, pharmaceutical companies should pay more attention to the demographic characteristics of physicians (age and sex) and their advertisement and pricing strategies.

## Introduction

Providing medicines in an accessible and affordable manner is the aim of all health systems. Establishment and reinforcement of local pharmaceutical manufacturing is one of the strategies to achieve mentioned aim, but viability and competitiveness of the local industry is necessary. In this way, local industry needs to know and understand the behavior of the target market and factors affecting selection of a pharmaceutical product by customers. On the other hand, understanding effects of different effective factors can be useful to optimize promotion activities which in turn can lead to the cost reduction.

*Iranian pharmaceutical context*

The year 1981 witnessed the beginning of a roundup of actions aimed at adopting and implementing policies to modernize the Iranian pharmaceutical sector, which influenced this industry all the way up to 1994 (1). Analysis of the Iranian pharmaceutical market in the 13-year period shows an annual sale growth equal to 28.38%. A study on domestic production and import revealed 9.3% and 42.3% annual growth, respectively (2). Mentioned actions, entitled Generic Scheme, sometimes also called the Generic Concept, formed the foundation of the new pharmaceutical system in the country. In recent years, national pharmaceutical system has been directed to the brand-generic and brand systems. This policy increased competition in pharmaceutical industry (3). The population of Iran is now over 74 million. The country’s gross domestic product (GDP) per capita in 2011 was reported to be over US$12,000 and the country spends about 6% of its GDP on health (4). 

*Literature review*

Physicians and patients have principal-agent relationship that arises under conditions of imperfect information (5). As agents, physicians play main role in deciding which medication or method of treatment best fits the patients’ health condition (6, 7). Many factors affect medication prescribing, including pharmaceutical industry influences, academic detailing interventions, efforts to educate health care providers, personal experience with a medication or class of medications, and patient requests (8). The competition between pharmaceutical companies in selling their products in domestic and international markets has caused huge investment in developing marketing strategies with direct focus on physicians and in some territories, patients (9, 10). 

Pharmaceutical companies seek for the best strategies in their targeted markets regarding the physicians’ and patients’ attitude and market characteristics (11). Several studies have been conducted to assess factors affecting the sale of prescription drugs such as age and sex of prescriber, price, advertising (12, 13). Considering previous studies conducted on this subject, in this study we aimed to assess the effects of five main factors, including: advertisement, insurance coverage, price, gender and age of prescriber on drug selling in Iran health market (12, 14).

*Data and variables*

In an effort to investigate the effect of price, advertisement and insurance coverage as well as characteristics of the physician (age and sex), the group of Antidepressant drugs was selected and from each a good sold drug whose data was accessed in Iran was chosen. The drug was Fluvoxamine.

We selected Fluvoxamine because of being new which facilitated the evaluation of the effect of age and sex in the physicians’ acceptability of the new drug. Both of the domestic generic and the imported brand product (Luvox®) are available in Iran drug market. Therefore we could analyze the effects of advertisement and insurance covarage on acceptability of a drug by physicians.

In order to evaluate the effect of advertisement on prescription, we compared generic and brand (Luvox®) types in Iranian drug market. We have assumed that the importer company uses advertising for brand type, but for generic type domestic producers don’t. The domestic generic type is covered by insurance but the brand form isn’t so we could examine the insurance coverage and ads effect.

## Experimental

*Method*

In order to find out the effect of each factor on the rate of prescription of each medicine, a multivariate model was offered and the impact of each component was studied within that model. 

*Statistical method*

According to the model offered in this study, the data of the drug prescribed by physicians were gathered in the time period between 2007 & 2009 and combination data method in econometrics (panel data) was used for estimation of the model.

Panel (data) analysis is used in epidemiology, econometrics and social science, which deals with two-dimensional (cross sectional/times series) data. In other words, the data related to each case are usually collected over the time and then a regression is run over these two dimensions. Multidimensional analysis is an econometric method in which data are collected over more than two dimensions (typically, time, individuals, and some third dimension). 

A common panel data regression model looks like *y*_it _*= a + bx*_it_* +Є*_it_, where *y* is the dependent variable, *x* is the independent variable, *a* and *b* are coefficients, *i* and *t* are indices for individuals and time. The error term, *Є*_it__,_ is very important in this analysis. Assumptions about the error term determine whether we speak of fixed effects or random effects. In a fixed effect model, *Є*_it_ is assumed to vary non-stochastically over *i* or* t* making the fixed effects model analogous to a dummy variable model in one dimension. In a random effects model, *Є*_it_ is assumed to vary stochastically over *i* or* t* requiring special treatment of the error variance matrix (16). Panel data sets for economic research possess several major advantages over conventional cross-sectional or time-series data sets (17, 18).

First, panel data usually give the researcher a large number of data points (n_i_ * m_t_), increasing the degrees of freedom and reducing the collinearity among explanatory variables.

Second and more importantly, longitudinal data allow a researcher to analyze a number of important economic questions that cannot be addressed using cross-sectional or time-series data sets.

Third, panel data provides a means of resolving the magnitude of econometric problems that often arises in empirical studies, namely the often heard assertion that the real reason one finds (or does not find) certain effects is the presence of omitted (miss measured or unobserved) variables that are correlated with explanatory variables (19). Whereas both brand and generic types of the drugs are available, two models, namely a generic and a brand were performed. Models were estimated using Eviews 6.0 software package.

Our sample includes 200 physicians who prescribed Fluvoxamine between 2007 & 2009. 

In this study, antidepressant drug categories were chosen because: First, these drugs are often used for chronic diseases so we can measure the impact of various factors over the long-term time frame. Second, there is no strong clinical evidence that the various antidepressants have different rates of efficacy. Thus, these drugs can be replaced with other antidepressant drugs category so the impact of other factors such as price and advertisement can be better measured (20, 21).

The antidepressant drug, Fluvoxamine was chosen because at the time the study was conducted, both generic and brand types, were available. Also this drug in the pharmaceutical market of Iran was new. Therefore, we could measure the response of physicians to new medicines. We calculated that each doctor how often prescribed this medication. Data were taken from the Social Security Organization of Iran (SSOI). The amount of money that is spent annually for advertising the drug, was obtained from importing company. 

The proposed model follows hereunder. Factors affecting the sale of prescribed drugs in Iran were analyzed using this model. Variables of the model are explained in Table1.

Y_ijt_ = F (P_jt_, M_t_, AD_jt_, D_1_, D_2_)

**Table 1:** Definitions of variables

**Variable name**	**Definition**
Y_ijt_	the amount of the sale of prescribed drug j by physician *i* in time *t*
P_jt_	the price of prescribed drug *j* in time *t*
M_t_	the age of physician *i *in time *t*
AD_jt_	the amount of advertisement of the prescribed drug j in time t
D1	the virtual variable indicating the coverage or non-coverage of drugs by insurance
D2	the virtual variable indicating the Physician gender

## Results

In order to investigate the factors affecting the prescription of the generic and brand (The brand type is not covered by insurance) of the drug two models are estimated as follows: according to the Table 2, the coefficients of all independent variables are statistically significant. There is a positive relation between the age and frequency of prescription of generic product and positive relation between male gender of the physician and the frequency of prescription of this product too. The estimated model for brand Fluvoxamine which enjoys advertisement is summarized in Table 3. There is a negative relation between the age of the physician and the frequency of prescription of the drug whereby older physicians prescribe the brand type less than younger ones. As we can see in the results, advertisements, have a positive impact on the prescription. 

Increasing price have negative effect in both generic and brand type. 

**Table 2:** Results of panel data test with Eviews 6.0 for Fluvoxamine

**Variable**	**Coefficient**	**t-static**		**Prob**
**Intercept**	27352.89	0.38		0.70
**Gender**	360.5	2.44		0.01
**Age**	0.0073	4.85		0.00
**Price**	-79	0.328		-0.74
	R^2^=0.904		R^2^=0.901	

**Table 3:** Results of panel data test with Eviews 6.0 for Luvox.

**Variable**	**Coefficient**	**t-static**		**Prob**
**Intercept**	68041.1	46.94		0.00
**Gender**	-525	-1.37		0.17
**Age**	-0.0327	-2.61		0.00
**Price**	-391.4	-68.8		0.00
**Advertisement**	0.000123	67.3		0.00
**Insurance**	68041.1	46.94		0.00
	R^2^=0.716		R^2^=0.706	

## Discussion

The results of the estimation imply that foreign brand (Luvox) advertisements had a positive and significant effect on the sale of this drug. From quantitative perspective it can be concluded that a marginal increase equal to one Rial (Iran currency) in advertising the drug, is associated with an increase equal to 0.000123 in the number of prescriptions for this drugs. In other words, spending one million Rials in advertising to the doctors, leads to one hundred and twenty-three additional prescriptions.

As the results show, gender and age of physicians had a significant effect on the frequency of prescription of the generic product. As can be seen from Table 2, male gender has a positive effect which may be interpreted, for Flovoxamine as a new drug in Iran market; male doctors are more inclined to prescribe new drugs.

For the brand type age had a negative and significant effect that means younger doctors more willing to prescribe brand type. In case of the responsiveness of prescription to the prices we should say that there is a statistically negative and significant relation between the prices of the generic and brand type of the drug and the frequency of its prescription. The results show at least 69 numbers reduction in the number of prescriptions for one Rial increase to the price. It should be noted that Fluvoxamine as an antidepressant drug should be used for a long time. Therefore elasticity of the price increases and physician is more willing to prescribe less expensive drug. In Iran health market, since the brand type is much more expensive than generic type, doctors are more sensitive to price changes in brand type. Insurance has a positive effect but as the result shows (Table 3) its effect is very high compared to other factors. 

The results of this study are consistent with previous studies which have estimated the positive effect of different kinds of advertisement strategies on prescription frequency by physicians (22-25). There are also studies about the effect of price and price-affecting factors -including health insurance coverage with the similar result of this study (26). The effect of different approach on pricing regulation has also been the subject of many studies (27).

Empirical evidence shows that insurance coverage is associated with rising health expenditure (28, 29). Because doctors’ prescriptions are a major source of health expenditures, exploring whether and why doctors respond to patients’ insurance is essential for understanding why expanding insurance coverage leads to rising expenditures. There are some studies which show it is more likely that a doctor prescribes brand type drugs or more expensive drugs to patients with insurance compared to patients without insurance (30, 31). 

There are also many articles about the effect of the gender of consumers and providers on the amount of the sale of a product. Most of the works have been carried out about women as customers of a particular series of products and shows that men are more independent, more certain, competitive, enthusiastic to change and risk (32). Similarly about physicians, a study conducted on 358 women and men showed that male physicians pay more attention to new technologies than female physicians therefore prescribe newer drugs (33). Stevenson and Tamblyn conclude in a qualitative report that female physicians principally prescribe fewer drugs, carry out less diagnostic activities and tend to be more favorable toward prevention of drug consumption (34).

About the age, studies have indicated that older physicians are less willing to use the newer drugs (35). It has been shown the year of graduation from university is an effective factor in prescription (36).

*Limitation*

In this study we were faced with some limitation. We had problem with data collection because cost of companies’ promotion for each product was not clear. Also due to the lack of prescription data of all insurer companies, we assumed that SSOI insurer data can be extended to the whole insurance information.
